# The association between recommended and non-recommended food scores on cardiovascular risk factors in obese and overweight adult women: a cross-sectional study

**DOI:** 10.1186/s12889-021-12404-1

**Published:** 2022-04-21

**Authors:** Maryam Sabbari, Atieh Mirzababaei, Farideh Shiraseb, Cain C. T. Clark, Khadijeh Mirzaei

**Affiliations:** 1 Faculty of Medical Sciences and Technologies, Islamic Azad University, Science and Research Branch of Tehran, Tehran, Iran; 2 Department of Community Nutrition, School of Nutritional Sciences and Dietetics, Tehran University of Medical Sciences (TUMS), P.O. Box: 14155-6117, Tehran, Iran; 3 Centre for Intelligent Healthcare, Coventry University, Coventry, CV1 5FB UK

**Keywords:** RFS, NRFS, Cardiovascular risk factors, Obesity

## Abstract

**Objective:**

Obesity is a highly prevalent, non-communicable, disease associated with numerous comorbid complications, such as cardiovascular disease. Following a healthy diet is known to help reduce the risk of both obesity and cardiovascular disease. This study was conducted to evaluate the association of recommended food score (RFS) and none recommended food score (NRFS) with cardiovascular risk factors in overweight and obese women.

**Methods:**

This cross-sectional study was performed on 379 overweight and obese (BMI ≥25 kg/m^2^) women aged 18-48 years. Anthropometric measurements and body composition analysis were assessed in all participants. Dietary intake was assessed by a valid and reliable food frequency questionnaire (FFQ) containing 147 items, and RFS and NRFS was calculated. Biochemical assessments including TC, HDL, LDL, TG, FBS, insulin, HOMA-IR, and hs-CRP were quantified by ELISA.

**Results:**

The mean age and BMI of participants were 36.73 ± 9.21 (y) and 31.17 ± 4.22 (kg/m^2^), respectively. Binary logistic regression showed that participants in the highest tertile of the RFS compared to the lowest tertile had 57% lower odds for hypertriglyceridemia [OR = 0.43, 95%CI = 0.20-0.92, *P* = 0.03]. Subjects with high adherence to the NRFS had lower HDL [OR = 2.11, 95%CI = 1.08-4.12, *P* = 0.02] and higher odds for hypertriglyceridemia [OR = 2.95, 95%CI = 1.47-5.94, *P* = 0.002] compared to low adherence.

**Conclusions:**

There was an inverse significant association between adherence to RFS and odds of hypertriglyceridemia. There was a significant association between NRFS and hypertriglyceridemia, in addition to an inverse association between NRFS and HDL. We recommend that people increase their consumption of fruits, vegetables, whole grains, lean meats or meat alternates, and low-fat dairy and avoid red meat, processed meat, chips, high-fat dairy, solid oil, refined grains, and variety of sweetened foods to prevent cardiovascular disease.

## Introduction

Currently, one third of the world’s population is overweight or obese, and it is projected that this incidence will increase to 57.8% by 2030obese [1]. Recent estimates indicate that the prevalence of obesity in Iran is increasing and may now be more than 26% of the population. In addition, its’ prevalence is higher in Iranian women vs. men [2], which may be attributed to differences between sex hormones in men and women and lower resting metabolic rate (RMR) in women [3]. Obesity negatively affects almost all physiological functions of the body and increases blood pressure (BP) [4], blood sugar [5], triglyceride (TG), and low-density lipoprotein cholesterol (LDL-C), and decreases high-density lipoprotein cholesterol (HDL-C) [6]. These changes increase the risk of cardiovascular disease (CVD). and it has been shown that obesity is an independent risk factor for CVD ( [7, 8]). The etiology of obesity is complex and multifactorial, and arises from the interaction of genetic, physiological, environmental, psychological, social, and economic factors. Indeed, among these factors, diet plays an important role in the development of both obesity and CVD ( [9, 10]).

Many methods have been proposed to evaluate diet quality; in some methods, the amount of single nutrients is assessed, whilst there are various indicators that focus on total diet or food groups. One approach to evaluating dietary patterns is to separate good and bad foods to describe a “healthy diet” and a “less healthy diet” [11]; recommended food score (RFS) [12] and non-recommended food score (NRFS) [13] were developed on this basis. RFS includes fruits, vegetables, whole grains, lean meats or meat alternates, and low-fat dairy [12]; whilst NRFS includes red meat, processed meat, chips, high-fat dairy, solid oil, refined grains, and variety of sweetened foods [13].

Numerous studies have reported the beneficial effects of adhering to diets rich in whole grains or fruits and vegetables on weight management and cardiovascular risk factors ( [14, 15]). These diets tend to be high in fiber, folate, nitrate, vitamins, and flavonoids and these compounds may reduce oxidative stress and modify lipid levels [16]. The extant literature suggests that women with higher RFS have lower mortality [12], particularly lower coronary heart disease (CHD) and stroke mortality. It has also been observed that adherence to the dietary approaches to stop hypertension (DASH) diet, which is high in fruit, vegetables, and low-fat dairy foods, significantly lowers BP, LDL [17], TG [18], high-sensitivity C-reactive protein (hs-CRP), and increases HDL [19]. In addition, although NRFS has been suggested not to play an important role in mortality from cancer, CHD, and stroke [13], high consumption of red and processed meat is known to raise BP and LDL ( [20, 21]). Indeed, some studies have shown that consuming high-fat dairy products increases LDL [22], and that the consumption of high- carbohydrate foods with high glycemic indices (GI) increases glucose, homeostatic model assessment insulin resistance (HOMA-IR), and insulin levels [23]. Given that there is no study that has clarified the association between RFS or NRFS and cardiovascular risk factors, we sought to evaluate the association of recommended food score (RFS) and non-recommended food score (NRFS) with cardiovascular risk factors in overweight and obese women. We hypothesized that RFS and NRFS would be associated with cardiovascular risk factors.

## Method

### Study population

The present cross-sectional study was performed using 379 obese or overweight women, who were randomly selected from individuals referred to health centers in Tehran. Inclusion criteria were being female, aged 18–48 years, and body mass index (BMI) ≥25 kg/m^2^. Exclusion criteria included; presence of cancer, liver or kidney disease, thyroid disease, other acute and chronic diseases, smoking, taking weight loss supplements, use of drugs to lower blood sugar, blood pressure and blood lipids, use of alcohol, pregnancy or lactation, or adherence to a specific diet over the past year. We also excluded patients who reported a total energy intake outside the range of 800–4200 kcal/day. The protocol was approved by ethics committee of Tehran University of Medical Sciences (IR.TUMS.VCR.REC.1397.577). All protocols were carried out in accordance with relevant guidelines and all participants signed an informed consent form.

### Dietary assessment

To assess the dietary intake of participants, a 147- item semi-quantitative food frequency questionnaire (FFQ) was used. The validity and reliability of FFQ has been approved in Iran [24].The FFQ evaluates the usual food intake over the previous year and consists of a list of foods with standard serving sizes usually consumed by Iranians. We used FFQ in previous studies and have described it in detail elsewhere [25]. All FFQ questionnaires were completed by trained dietitians during face-to-face interviews. Food analysis was conducted using Nutritionist IV software, modified to reflect the Iranian context (First Databank Division, The Hearst Corporation, San Bruno, CA, USA).

### Recommended food score and non-recommended food score

The RFS was developed by Kant et al. to measure overall diet quality, and is based on the consumption of foods recommended by dietary guidelines [12]. We rearranged RFS based on the Iranian diet, so some of its components are different from the RFS provided by Kant et al. The RFS included the following foods: apples or pears; oranges; cantaloupe; grapefruit; orange or grapefruit juice; other fruit juices; tomatoes; broccoli; spinach; turnip; carrots; green vegetables; potatoes; baked or stewed chicken; baked or broiled fish; beans; whole wheat bread; dark toast; low fat milk; low fat yogurt. The RFS is calculated by summing that the items consumed at least once a week, so the maximum score is 20. NRFS was developed by Michels et al. as an adjunct to RFS [13]. We also rearranged NRFS based on the Iranian diet, where our NRFS included: meat; beef; minced meat; liver/kidney; bacon/ sausages; cold cuts; fried potatoes; chips; high fat milk/ yogurt; cheese; ice cream; cream; butter/margarine; hydrogenated vegetable oil; white bread; spaghetti; sugar; candy; biscuits. Table 1 shows the components of RFS and NRFS. All dietary components were adjusted for energy. For each food item that was consumed at least once a month, a score of 1 was given, and a maximum score of 19 was possible.

**Table 1:** Foods and food groups in RFS and NRFS

Food items	
RFS	NRFS
apple or pear	Meat
Orange	Beef
Grapefruit	minced meat
Cantaloupe	liver/kidney
orange or grapefruit juice	bacon/ sausages
other fruit juice	Cold cut
Tomato	High fat milk/ yogurt
Broccoli	Cheese
Spinach	Ice cream
Turnip	Cream
Carrot	butter/margarine
Green vegetable	hydrogenated vegetable oil
Potato	fried potatoes
baked or stewed chicken	Chips
baked or broiled fish	white bread
Beans	Spaghetti
whole wheat bread	Sugar
dark toast	Candy
low fat milk	Biscuits
low fat yogurt	

### Biochemical assessment

After 12 to 14 h of overnight fasting, blood samples were drawn from all participants. Serum samples were centrifuged for 10 min at 3000 rpm, divided into 1 ml tubes, and were frozen at − 80 °C. Serum concentrations of total cholesterol (TC), high-density lipoprotein cholesterol (HDL-C), low-density lipoprotein cholesterol (LDL-C), and triglyceride (TG) were evaluated by using of enzymatic approaches using related kits (Pars Azmun, Iran) and auto analyzer system. The serum fasting glucose concentration was measured using an enzymatic colorimetric method with the glucose oxidase technique and Insulin level was assessed using the enzyme linked immunosorbent assay (ELISA) kit (Human insulin ELISA kit, DRG Pharmaceuticals, GmbH, Germany). Serum high-sensitive C-reactive protein (hs-CRP) was evaluated with the use of the immunoturbidimetric assay. All blood analyses were conducted at the Endocrinology and Metabolism Research Institute (EMRI) Bio nanotechnology laboratory of Tehran University of Medical Science.

### The HOMA-IR calculation

IR was calculated by the homeostatic model assessment (HOMA) method, according to the following equation: HOMA-IR = [fasting plasma glucose (mmol/l) × fasting plasma insulin (mIU/l)]/22.5 [26].

### Resting metabolic rate (RMR) measurement

The RMR was determined using indirect calorimetry based on the manufacturers’ protocol. Indirect calorimetry calculates the RMR by measuring the amount of consumed oxygen and produced carbon dioxide. The amount of inhaled and exhaled breath was transmitted by a filter attached to the mask that completely covered a person’s nose and mouth, and sensor. The device measured the concentration of CO_2_ and O_2_ using the ventilated hood and analyzed the RMR. All measurements were assessed in the morning, after a comfortable night’s sleep. Participants were instructed to fast, and drink only water, for 12 h before testing and wear comfortable clothing and refrain from any severe-intensity physical activity [27].

### Anthropometric assessment

Height was measured, while participants were standing, unshod, with their shoulders in a normal position, using a stadiometer (Seca, Hamburg, Germany), and was recorded to the nearest 0.5 cm. Next, while subjects were minimally clothed and unshod, weight was measured with the use of a digital scale (Seca, Hamburg, Germany) and recorded to the nearest 100 g. Obesity and overweight were defined as BMI ≥ 30 kg/m^2^ and 25 ≤ BMI ≤ 29.9 kg/m^2^, respectively. BMI was calculated as weight divided by height squared (kg/m^2^).

### Body composition analysis

Body composition parameters included amount and proportion of body fat percentage (BF %), fat mass (FM) and fat free mass (FFM), waist circumference (WC) and waist-to-hip ratio (WHR) were taken by multi-frequency bioelectrical impedance analyzer (BIA): InBody 770 Scanner (InBody Co., Seoul, Korea). Measurements were performed in the morning in a fasted state with light clothing. Participants were asked not to exercise, not to carry any electric devices, and to urinate just before the body composition analysis, to yield a more accurate result. According to manufacturer instructions, participants stood on the scale in bare feet and held the handles of the machine for 20 s, then, the output was printed. The precise measurement method has been described in detail elsewhere [27].

### Assessment of blood pressure

Blood pressure and pulse were measured using a standard sphygmomanometer (Omron, Germany, European) by a trained physician, while the participants were at rest for 15 min. Hypertension was defined as systolic blood pressure ≥ 130 mmHg or diastolic blood pressure ≥ 85 mmHg [28].

### Assessment of other variables

The International Physical Activity Questionnaire (IPAQ), that was calculated as metabolic equivalent hours per week (METs h/week), was used to assess physical activity (PA) [29]. The PA of the participants was classified as follows: low < 600 (METs h/week), moderate = 600-3000 (METs h/week) and severe > 3000 (METs h/week) [30].

Demographic characteristics including age, marital condition, education status, particular diets, chronic disease history, and medicine consumption were asked by a trained nutritionist.

### Statistical analysis

Normality distribution was evaluated by applying the Kolmogorov-Smirnov’s test. For describing the baseline characteristics of the study population, descriptive analysis was used. Data pertaining to quantitative characteristics were reported as the mean ± standard deviation (SD) and data regarding qualitative characteristics were expressed as a number. Score indicating adherence to the RFS and NRFS, respectively, were calculated. All subjects were ranked according to their scores to the 3 RFS and NRFS groups. One-way Analysis of variance (ANOVA) and Chi-square tests were used to compare quantitative and qualitative characteristics of participants across different values of adherence to the RFS and NRFS. Formal tests for interaction were performed, but without significant results. To determine the association between RFS and NRFS and cardiovascular risk factors, logistic binary regression was utilized, in a crude and adjusted model. Adjustments were made for age [31], energy [32], PA ( [33, 34]), BMI [35], RMR [36], education level [37], marital status [38], diet resistance [39], age of onset of obesity [40], Family history of obesity [41], and economic status [33, 37, 42, 43]. We selected these confounders based on previous studies and considered the items as related to our outcomes and exposures. In all multivariate models, T1 of the RFS and NRFS was considered as reference. Statistical analysis was performed using SPSS v23 software, whilst a *P*-value less than 0.05 was defined as the representing statistical significance, a priori.

## Results

### Study population

The mean age, weight, and BMI of participants were 36.73 ± 9.21 (y), 80.94 ± 12.08 (kg), and 31.17 ± 4.22 (kg/m^2^) respectively. The biochemical, anthropometric and demographic characteristics of the subjects are reported across the RFS tertiles in Table 2. In the crude model, continuous variables were compared using ANOVA and categorized variables were compared using Chi-square tests across the RFS tertiles. In the adjusted model, variables were compared using ANCOVA. After adjustment, a significant difference in distribution of TG (*P* = 0.01) across RFS groups was observed. Other variables did not significantly differ between the RFS tertiles.

**Table 2:** Participant characteristics in RFS tertiles

Variables	RFS			*P* value*	P value**
	T1 (*n* = 126)	T2 (n = 126)	T3 (n = 126)		
	mean ± SD				
Demography					
Age(y)	36.34 ± 9.03	36.44 ± 9.53	37.41 ± 9.09	0.51	0.21^a^
Weight(kg)	80.54 ± 12.65	81.43 ± 11.90	80.84 ± 11.75	0.84	0.45
Height(cm)	161.09 ± 5.87	161.84 ± 6.12	161.63 ± 5.66	0.26	0.44
Blood pressure					
SBP(mmHg)	111.71 ± 16.77	111.83 ± 13.99	110.27 ± 13.37	0.73	0.11
DBP(mmHg)	76.60 ± 12.54	78.40 ± 9.65	77.51 ± 8.76	0.50	0.33
Pulse	81.39 ± 11.82	79.22 ± 10.64	78.17 ± 8.68	0.12	0.44
RMR	1587.46 ± 273.41	1588.12 ± 256.11	1556.59 ± 254.07	0.65	0.81
Body composition					
BFM(kg)	34.55 ± 8.77	34.62 ± 8.41	34.43 ± 8.71	0.98	0.55^b^
FFM(kg)	46.11 ± 5.89	47.17 ± 5.66	46.11 ± 5.41	0.23	0.38^b^
SMM(kg)	25.38 ± 3.65	25.92 ± 3.37	25.26 ± 3.21	0.25	0.35^b^
BMI (kg/m^2^)	31.02 ± 4.32	31.23 ± 4.15	31.25 ± 4.23	0.89	0.23^b^
PBF(%)	42.32 ± 5.18	41.97 ± 5.20	42.05 ± 6.10	0.86	0.84^b^
WHR	0.93 ± 0.05	0.94 ± 0.05	1.65 ± 8.11	0.37	0.53^b^
WC(cm)	99.30 ± 10.38	100.19 ± 9.88	98.80 ± 9.69	0.53	0.30^b^
Biochemical assessment					
FBS(mg/dl)	88.18 ± 11.80	87.23 ± 8.33	86.67 ± 8.24	0.60	0.17
T-Chol (mg/dl)	184.92 ± 38.85	187.53 ± 35.18	183.00 ± 33.26	0.73	0.27
HDL(mg/dl)	48.00 ± 10.19	45.28 ± 11.03	47.48 ± 11.48	0.25	0.45
LDL(mg/dl)	93.44 ± 23.31	96.43 ± 26.61	95.88 ± 22.44	0.70	0.65
TG(mg/dl)	118.14 ± 60.51	114.90 ± 55.46	120.90 ± 61.06	0.81	0.01
ALT(U/L)	18.31 ± 13.14	21.22 ± 14.50	18.30 ± 11.32	0.27	0.38
AST(U/L)	17.24 ± 6.92	18.63 ± 8.44	17.94 ± 6.83	0.49	0.51
Hs.CRP(mg/L)	3.70 ± 4.11	4.17 ± 4.88	4.93 ± 4.77	0.24	0.07
HOMA-IR	3.25 ± 1.29	3.38 ± 1.27	3.33 ± 1.24	0.84	0.97
Insulin(IU/ml)	1.23 ± 0.25	1.17 ± 0.19	1.23 ± 0.23	0.15	0.47
Qualitative variables ^€^					
PA(METs h/week)				0.82	0.53^c^
Low	43(35.5)	42(34.7)	36(29.8)		
Moderate	33(29.2)	39(34.5)	41(36.3)		
High	4(33.3)	4(33.3)	4(33.3)		
Marital status				0.21	0.12
Single	38(37.3)	37(36.3)	27(26.5)		
Married	86(31.3)	90(32.7)	99(36.0)		
Education				0.70	0.15
Illiterate	1(25.0)	1(25.0)	2(50.0)		
Diploma	15(31.3)	20(41.7)	13(27.1)		
Bachelor and higher	108(33.2)	106(32.6)	111(34.2)		
Economic status				0.78	0.59
Poor	14(36.8)	15(39.5)	9(23.7)		
Moderate	50(31.1)	53(32.9)	58(36.0)		
Good	49(33.3)	46(31.3)	52(35.4)		
Rich	6(31.6)	8(42.1)	5(26.3)		
History of weight loss				0.94	0.86
Yes	62(32.6)	66(34.7)	62(32.6)		
No	53(33.8)	52(33.1)	52(33.1)		
Resistant to diet				0.43	0.59
Yes	30(32.6)	26(28.3)	36(39.1)		
No	85(34.0)	87(34.8)	78(31.2)		

¥: Data are presented as Mean ± SD. €: Data are presented as n (%). Abbreviations: PA: physical activity; SBP: systolic blood pressure; DBP: diastolic blood pressure; RMR: resting metabolic rate; BFM: body fat mass; FFM: fat free mass; SMM: Skeletal muscle mass; BMI: body mass index; PBF: Percent body fat; WHR: Waist hip ratio; WC: Waist circumference; FBS: free blood sugar; HDL: high-density lipoprotein; LDL: low-density lipoprotein; ALT: Alanine aminotransferase; AST: aspartate aminotransferase; hs-CRP: high sensitivity C-reactive protein. HOMA-IR: Homeostatic Model Assessment for Insulin Resistance

*P values resulted from ANOVA analysis. P value < 0.05 is significant

**P values presented resulted from ANCOVA analysis and were adjusted for age, BMI, energy and physical activity

a: age considered as collinear and this variable adjusted for BMI, energy and physical activity

b: BMI considered as collinear and this variable adjusted for age, energy and physical activity

c: PA considered as collinear and this variable adjusted for age, BMI and energy

Table 3 presents the characteristics of the participants by tertiles of NRFS. Continuous variables were compared using ANOVA and categorized variables were compared using Chi-square tests across the NRFS tertiles. Our findings showed a marginal significant difference in distribution of RMR (*P* = 0.05) and a significant difference in distribution of economic status (P = 0.03) across NRFS groups, but after adjustment, these differences disappeared. Other variables did not significantly differ between the NRFS groups.

**Table 3:** Participant characteristics in NRFS tertiles

variables	NRFS			P value*	P value**
	T1 (n = 126)	T2 (*n* = 127)	T3 (n = 126)		
	mean ± SD				
Demography					
Age(y)	36.36 ± 9.67	37.01 ± 8.97	36.82 ± 9.02	0.84	0.60^a^
Weight(kg)	81.37 ± 12.25	79.16 ± 11.05	82.29 ± 12.76	0.10	0.54
Height(cm)	161.51 ± 5.33	160.53 ± 6.50	161.52 ± 5.78	0.30	0.80
Blood pressure					
SBP(mmHg)	109.23 ± 15.66	112.70 ± 14.76	111.83 ± 13.93	0.26	0.43
DBP(mmHg)	75.95 ± 11.24	78.00 ± 9.66	78.43 ± 10.52	0.24	0.24
Pulse	79.57 ± 11.32	79.75 ± 9.24	79.67 ± 11.18	0.99	0.92
RMR	1595.51 ± 237.09	1526.38 ± 257 ± 05	1612 ± 16,280.07	0.05	0.89
Body composition					
BFM(kg)	34.87 ± 8.69	33.67 ± 7.75	35.06 ± 9.31	0.37	0.20^b^
FFM(kg)	46.64 ± 5.39	45.77 ± 5.94	46.99 ± 5.63	0.21	0.90^b^
SMM(kg)	25.59 ± 3.22	25.17 ± 3.67	25.81 ± 3.34	0.32	0.83^b^
BMI(kg/m^2^)	31.21 ± 4.35	30.80 ± 3.76	31.49 ± 4.52	0.42	0.61^b^
PBF(%)	42.30 ± 5.10	42.06 ± 5.28	41.98 ± 6.09	0.89	0.18^b^
WHR	1.66 ± 8.11	0.93 ± 0.05	0.93 ± 0.05	0.36	0.65^b^
WC(cm)	99.88 ± 10.41	98.37 ± 9.21	100.05 ± 10.27	0.34	0.48^b^
Biochemical assessment					
FBS(mg/dl)	86.02 ± 7.21	87.66 ± 11.75	88.24 ± 9.11	0.34	0.41
T-Chol (mg/dl)	185.50 ± 40.62	182.42 ± 32.36	187.49 ± 35.14	0.65	0.78
HDL(mg/dl)	46.77 ± 12.27	48.36 ± 10.64	45.75 ± 9.91	0.29	0.29
LDL(mg/dl)	94.67 ± 27.48	94.19 ± 22.37	96.61 ± 22.89	0.79	0.78
TG(mg/dl)	114.85 ± 55.03	125.75 ± 56.20	114.53 ± 67.33	0.41	0.36
ALT(U/L)	18.17 ± 6.88	17.19 ± 6.29	18.42 ± 8.74	0.52	0.66
AST(U/L)	19.07 ± 10.58	18.42 ± 12.74	20.17 ± 15.13	0.68	0.80
Hs.CRP (mg/L)	4.04 ± 4.04	4.04 ± 4.85	4.64 ± 4.76	0.64	0.29
HOMA-IR	3.38 ± 1.13	3.44 ± 1.49	3.09 ± 1.15	0.25	0.10
Insulin(IU/ml)	1.21 ± .24	1.24 ± .23	1.18 ± .19	0.23	0.14
Qualitative variables ^€^					
PA (METs h/week)				0.54	0.36^c^
Low	38(31.4)	47(38.8)	36(29.8)		
Moderate	34(30.1)	34(30.1)	45(39.8)		
High	4(33.3)	4(33.3)	4(33.3)		
Marital status				0.20	0.58
Single	41(40.2)	30(29.4)	31(30.4)		
Married	84(30.5)	96(34.9)	95(34.5)		
Education				0.83	0.45
Illiterate	1(25)	2(50)	1(25)		
Diploma	14(29.2)	15(31.3)	19(39.6)		
Bachelor and higher	110(33.8)	109(33.5)	106(32.6)		
Economic status				0.03	0.35
Poor	8(21.1)	18(47.4)	12(31.6)		
Moderate	61(37.9)	51(31.7)	49(30.4)		
Good	42(28.6)	47(32)	58(39.5)		
Rich	10(52.6)	7(36.8)	2(10.5)		
Resistant to diet				0.34	0.21
Yes	32(34.8)	35(38)	25(27.2)		
No	81(32.4)	76(30.4)	93(37.2)		
Family history of obesity				0.13	0.34
Yes	77(30.1)	93(36.3)	86(33.6)		
No	40(39.2)	27(26.5)	35(34.3)		

¥: Data are presented as Mean ± SD

€: Data are presented as n (%). Abbreviations: PA: physical activity; SBP: systolic blood pressure; DBP: diastolic blood pressure; RMR: resting metabolic rate; BFM: body fat mass; FFM: fat free mass; SMM: Skeletal muscle mass; BMI: body mass index; PBF: Percent body fat; WHR: Waist hip ratio; WC: Waist circumference; FBS: free blood sugar; HDL: high-density lipoprotein; LDL: low-density lipoprotein; ALT: Alanine aminotransferase; AST: aspartate aminotransferase; hs-CRP: high sensitivity C-reactive protein. HOMA-IR: Homeostatic Model Assessment for Insulin Resistance

*P values resulted from ANOVA analysis. P value < 0.05 is significant

**P values presented resulted from ANCOVA analysis and were adjusted for age, BMI, energy and physical activity

a: age considered as collinear and this variable adjusted for BMI, energy and physical activity

b: BMI considered as collinear and this variable adjusted for age, energy and physical activity

c: PA considered as collinear and this variable adjusted for age, BMI and energy

### Association between cardiovascular risk factors and RFS

The association between RFS tertiles and each of the cardiovascular risk factors in the crude model and adjusted model are reported in Table 4. To determine the association between RFS and cardiovascular risk factors, logistic binary regression was utilized in a crude model and adjusted model. We found that Participants who were in the highest tertile of the RFS compared to the lowest tertile had 57% lower odds for Hypertriglyceridemia [OR = 0.43, 95%CI = 0.20-0.92, P = 0.03]. However, there were no statistically significant differences in other cardiovascular risk factors included FBS, HDL, LDL, WC, HOMA-IR, and BP, among the RFS tertiles (*P* > 0.05).

**Table 4:** Association between RFS and cardiovascular risk factors

Variables	RFS			P trend
	T1	T2	T3	
FBS(mg/dl)				
Crude	1	1.20(0.72-2.00)	1.10(0.66-1.84)	
P value		0.46	0.69	
Model 1	1	2.01(0.69-5.79)	1.24(0.41-3.71)	0.86
P value		0.19	0.70	
T-Chol (mg/dl)				
Crude	1	1.23(0.74-2.03)	.96(0.58-1.59)	
P value		0.41	0.89	
Model 1	1	2.01(0.90-4.45)	0.99(0.45-2.21)	0.96
P value		0.08	0.99	
HDL(mg/dl)				
Crude	1	1.01(0.61-1.67)	0.96(0.58-1.59)	
P value		0.94	0.89	
Model 1	1	0.87(0.42-1.82)	0.99(0.48-2.06)	0.64
P value		0.72	0.99	
LDL(mg/dl)				
Crude	1	1.19(0.72-1.96)	1.03(0.62-1.70)	
P value		0.48	0.89	
Model 1	1	1.83(0.74-4.49)	1.01(0.40-2.59)	0.71
P value		0.18	0.97	
Homa-IR				
Crude	1	1.13(0.57-2.23)	0.76(0.40-1.45)	
P value		0.71	0.41	
Model 1	1	1.01(0.38-2.68)	0.42(0.17-1.05)	0.18
P value		0.98	0.06	
TG(mg/dl)				
Crude	1	0.83(0.50-1.37)	0.61(0.37-1.01)	
P value		0.48	**0.05**	
Model 1	1	0.88(0.40-1.90)	0.43(0.20-0.92)	**0.02**
P value		0.75	**0.03**	
WC(cm)				
Crude	1	1.00(0.47-2.16)	0.76(0.36-1.57)	
P value		0.98	0.46	
Model 1	1	0.37(0.06-2.33)	0.25(0.04-1.35)	0.84
P value		0.29	0.10	
Hypertension				
Crude	1	1.10(0.54-2.21)	0.79(0.37-1.66)	
P value		0.78	0.53	
Model 1	1	1.41(0.52-3.80)	0.76(0.27-2.14)	0.45
P value		0.49	0.61	

All values are presented as odds ratio (OR) and 95% Confidence intervals (95% CI)

Model 1: Adjusted for age, energy, physical activity, RMR, BMI, education, marriage, diet resistance, age at onset of obesity, Family history of obesity and socio economic status. *P* value < 0.05 is significant

Quartile 1 of recommended food score was considered as a reference group

### Association between cardiovascular risk factors and NRFS

Table 5 shows the association between cardiovascular risk factors and NRFS tertiles in two crude and adjusted models. To determine the association between NRFS and cardiovascular risk factors, logistic binary regression was utilized in a crude model and adjusted model. The results shown that Participants who were in the highest tertile of the NRFS compared to the lowest tertile had lower HDL [OR = 2.11, 95%CI = 1.08-4.12, P = 0.02]. Also the Participants who were in the highest tertile of the NRFS compared to the lowest tertile had higher odds for Hypertriglyceridemia [OR = 2.95, 95%CI =1.47-5.94, P = 0.002]. There were no statistically significant differences in other cardiovascular risk factors included FBS, LDL, WC, HOMA-IR, and BP, among the NRFS tertiles (P > 0.05).

**Table 5:** Association between NRFS and cardiovascular risk factors

Variables	Not recommended food score			P trend
	T1	T2	T3	
FBS(mg/dl)				
Crude	1	0.73(0.44-1.21)	0.67(0.40-1.11)	
P value		0.22	0.12	
Model 1	1	0.81(0.35-1.87)	0.59(0.25-1.36)	0.21
P value		0.63	0.22	
T-Chol(mg/dl)				
Crude	1	0.73(0.44-1.20)	0.82(0.49-1.35)	
P value		0.22	0.44	
Model 1	1	0.81(0.40-1.66)	0.80(0.40-1.61)	0.54
P value		0.58	0.54	
HDL(mg/dl)				
Crude	1	1.28(0.77-2.14)	1.68(1.02-2.79)	
P value		0.32	**0.04**	
Model 1	1	1.40(0.72-2.72)	2.11(1.08-4.12)	**0.02**
P value		0.32	**0.02**	
LDL(mg/dl)				
Crude	1	0.55(0.33-0.91)	0.57(0.35-0.95)	
P value		**0.02**	**0.03**	
Model 1	1	0.38(0.17-0.84)	0.47(0.22-0.99)	0.44
P value		**0.01**	0.44	
Homa-IR				
Crude	1	1.12(0.58-2.16)	1.05(0.55-2.01)	
P value		0.71	0.86	
Model 1	1	1.21(0.49-2.97)	0.78(0.34-1.79)	0.53
P value		0.67	0.56	
TG(mg/dl)				
Crude	1	1.99(1.20-3.28)	2.38(1.44-3.96)	
P value		**0.007**	**0.001**	
Model 1	1	2.78(1.38-5.60)	2.95(1.47-5.94)	**0.002**
P value		**0.004**	**0.002**	
WC(cm)				
Crude	1	0.82(0.40-1.69)	1.16(0.54-2.49)	
P value		0.60	0.69	
Model 1	1	0.70(0.16-3.03)	0.48(0.09-2.44)	0.38
P value		0.64	0.38	
hypertension				
Crude	1	1.56(0.73-3.30)	1.46(0.69-3.12)	
P value		0.24	0.31	
Model 1	1	1.90(0.78-4.63)	1.46(0.59-3.63)	0.44
P value		0.15	0.41	

All values are presented as odds ratio (OR) and 95% Confidence intervals (95% CI)

Model 1: Adjusted for age, energy, BMI, RMR, education, marriage, diet resistance, age at onset of obesity, Family history of obesity and socio economic status. *P* value < 0.05 is significant

Tertile 1 of NRFS was considered as a reference group

## Discussion

The results showed an inverse and significant association between adherence to RFS and odds of hypertriglyceridemia. Moreover, in this study, there was a significant association between NRFS and hypertriglyceridemia, in addition to an inverse association between NRFS and HDL. There was no statistically significant association between any other cardiovascular risk factor and RFS and NRFS.

Contrary to our results, a cross sectional study, including 1008 adults in Korea, found women with higher RFS and PA have lower risk of abdominal obesity [44]. In another cross-sectional study of Australian adults, it was observed, in men, that RFS was significantly inversely associated with systolic blood pressure (SBP) and diastolic blood pressure (DBP), but there was no association between RFS and BP in women. Concordant with our findings, in [45], RFS was not significantly associated with obesity in both men and women. Moreover, in a Prospective Cohort study of Korean Adults, who were followed from 2001 to 2014, it was observed the incidence of metabolic syndrome in the 5th RFS quintile group was significantly lower compared to the 1st quintile group after adjusting for age and energy intake in women; although after adjusting for additional covariates, this association disappeared [46].

There have been numerous reports pertaining to the effect of other healthy dietary patterns, such as DASH diet and Mediterranean diet, on cardiovascular risk factors, which are similar based on the consumption of fruits, vegetables, grains, dairy products, and fish. In a cross-sectional study, including 6874 older adults in Spain, participants with better adherence to the Mediterranean diet, compared with low adherence, had significantly lower average TG levels, BMI, and WC [47]. In another cross-sectional study conducted in Iran, being in the higher category of the Mediterranean diet score was associated with lower WC, TG, hs-CRP, and higher HDL-C. Also, adherence to the DASH diet was associated with lower DBP, insulin levels, and hs-CRP [48]. Evidently, following the DASH diet also lowers BP, which is because the DASH diet emphasizes reducing salt intake, but this is not measured in RFS. In contrast, however, in some clinical studies, the DASH diet had no effect on improving insulin sensitivity and TG ( [35, 36]).

RFS seems to be associated with reduced cardiovascular risk factors, such as TG, due to high amounts of fruits and vegetables, whole grains, and low-fat dairy products. Fruit and vegetables, which contain a wide range of potentially cardioprotective components, such as fiber, folate, nitrate, vitamins, and flavonoids. Dietary flavonoids act via different mechanisms of action to reduce cardiovascular risk factors. They can reduce oxidative stress, modify lipid levels, and regulate glucose metabolism [16]. Whole grains, fruits and vegetables are high in soluble and insoluble fiber; where soluble fiber can slow gastric emptying, increase satiety, and regulate cholesterol and blood sugar ( [2, 49]). The intestinal microflora ferments the indigestible carbohydrates in cereals into short-chain fatty acids (acetate, butyrate, and propionate), which are effective in reducing body weight, FBS, BP, and TG, and increasing HDL [2].

On the other hand, NRFS seems to be associated with increased cardiovascular risk factors, due to the high consumption of red and processed meats, saturated fats, refined carbohydrates, and a variety of sweetened foods. In a study conducted in Japan, participants who consumed high amounts of meat and fat had higher WC, BMI, BP, and blood lipid profile [50]. Although the results of some studies contradict this [51], the results of a meta-analysis showed that total, red, and processed meat intake is positively associated with metabolic syndrome [52]. Red meat contains high amounts of saturated fat and heme-iron; iron is a strong pro-oxidant, which can damage tissues such as pancreatic beta cells. Thus, a high iron level can impair glucose metabolism and decrease insulin levels ( [53, 54]). Additionally, nitrate, used as a preservative in processed meat, can be converted into nitrosamines, which have been shown to be toxic to pancreatic cells and lead to insulin resistance ( [55, 56]).

It has been observed that a diet high in sugar and refined carbohydrates is associated with increases TC, TG, LDL, the ratio of TC/HDL [57], glucose, HOMA-IR and insulin levels; in addition to increases in the expression of enzymes involved in fat synthesis, reductions in the expression of enzymes effective in lipolysis, and increases the accumulation of fat in the body [23]. In contrast, in another study conducted on Iranian women, diets lower in carbohydrate were not associated with overweight/obesity and cardiovascular risk factors [58].

This study possesses several strengths. Indeed, to our knowledge, this study is the first to show the relationship between RFS and cardiovascular risk factors in adult women. Moreover, the number of study participants was relatively high and known potential confounding factors were measured and controlled for in the analysis. Although the present study represents a novel addition to the literature, there are some limitations that should be considered. Due to the cross-sectional design, we could not evaluate causality between the RFS and cardiovascular risk factors. So, further prospective or interventionist research is needed to confirm whether the association truly represents a cause–effect relationship. The use of FFQs, although used widely, can result in under- or over-reporting of food intake, which should be acknowledged. Our study was conducted only on obese and overweight women, so we cannot extrapolate the results to the whole community. Finally, only the RFS was used to evaluate the dietary quality, and no instruments were used for assessing other nutrients [59].

.

## Conclusion

Overall, the results of our study show that adherence to RFS is inversely associated with hypertriglyceridemia, and there appears to be a direct link between NRFS and hypertriglyceridemia. Moreover, adherence to NRFS is also associated with decreased HDL**.** Although further prospective and clinical studies are needed, according to the results of this study, we advocate that people increase their consumption of fruits, vegetables, whole grains, lean meats or meat alternates, and low-fat dairy, and avoid/reduce red meat, processed meat, chips, high-fat dairy, solid oil, refined grains, and variety of sweetened foods, to help prevent cardiovascular disease.

## Data Availability

The datasets used and/or analyzed during the current study are available from the corresponding author on reasonable request.

## Abbreviations

RMR: resting metabolic rate
BP: blood pressure
TG: triglyceride
LDL: low-density lipoprotein
HDL: high-density lipoprotein
CVD: cardiovascular disease
RFS: recommended food score
NRFS: non-recommended food score
CHD: coronary heart disease
DASH: dietary approaches to stop hypertension
hs-CRP: high-sensitivity C-reactive protein
GI: glycemic index
HOMA-IR: Homeostatic model assessment insulin resistance
BMI: body mass index
FFQ: food frequency questionnaire
TC: total cholesterol
ELISA: enzyme linked immunosorbent assay
BF: fat percentage
FFM: fat free mass
FM: fat mass
WC: waist circumference
WHR: waist-to-hip ratio
BIA: bioelectrical impedance analyzer
IPAQ: International Physical Activity Questionnaire
PA: physical activity
OR: odds ratio
SBP: systolic blood pressure
DBP: diastolic blood pressure
